# Long-Term Exposure to Airborne Particles and Arterial Stiffness: The Multi-Ethnic Study of Atherosclerosis (MESA)

**DOI:** 10.1289/ehp.0901524

**Published:** 2011-01-18

**Authors:** Marie S. O’Neill, Ana V. Diez-Roux, Amy H. Auchincloss, Mingwu Shen, João A. Lima, Joseph F. Polak, R. Graham Barr, Joel Kaufman, David R. Jacobs

**Affiliations:** 1 University of Michigan School of Public Health, Ann Arbor, Michigan, USA; 2 Johns Hopkins University School of Medicine, Baltimore, Maryland, USA; 3 Tufts University–New England Medical Center, Boston, Massachusetts, USA; 4 Columbia University Medical Center, New York, New York, USA; 5 University of Washington School of Public Health, Seattle, Washington, USA; 6 University of Minnesota School of Public Health, Minneapolis, Minnesota, USA; 7 University of Oslo, Oslo, Norway

**Keywords:** air pollution, arterial stiffness, environmental air pollutants, epidemiology

## Abstract

**Background:**

Increased arterial stiffness could represent an intermediate subclinical outcome in the mechanistic pathway underlying associations between average long-term pollution exposure and cardiovascular events.

**Objective:**

We hypothesized that 20 years of exposure to particulate matter (PM) ≤ 2.5 and 10 μm in aerodynamic diameter (PM_2.5_ and PM_10_, respectively) would be positively associated with arterial stiffness in 3,996 participants from the Multi-Ethnic Study of Atherosclerosis (MESA) who were seen at six U.S. study sites.

**Methods:**

We assigned pollution exposure during two decades preceding a clinical exam (2000–2002) using observed PM_10_ from monitors nearest participants’ residences and PM_10_ and PM_2.5_ imputed from a space-time model. We examined three log-transformed arterial stiffness outcome measures: Young’s modulus (YM) from carotid artery ultrasound and large (C_1_) and small (C_2_) artery vessel compliance from the radial artery pulse wave. All associations are expressed per 10 μg/m^3^ increment in PM and were adjusted for weather, age, sex, race, glucose, triglycerides, diabetes, waist:hip ratio, seated mean arterial pressure, smoking status, pack-years, cigarettes per day, environmental tobacco smoke, and physical activity. C_1_ and C_2_ models were further adjusted for heart rate, weight, and height.

**Results:**

Long-term average particle exposure was not associated with greater arterial stiffness measured by YM, C_1_, or C_2_, and the few associations observed were not robust across metrics and adjustment schemes.

**Conclusions:**

Long-term particle mass exposure did not appear to be associated with greater arterial stiffness in this study sample.

Chronic exposure to elevated levels of particulate matter (PM) air pollution has been associated with cardiovascular mortality in several studies (Dockery et al. 1993; Hoek et al. 2002; Pope et al. 2002). However, mechanisms underlying these associations have yet to be fully elucidated, and understanding how long-term exposure to air pollution may affect cardiovascular health is an important public health goal. The primary candidate mechanisms (Pope et al. 2004) for which some preliminary evidence exists include air pollution influencing the development of atherosclerotic disease (Künzli et al. 2005), increasing vascular stiffness and impairing autonomic function (Brook et al. 2002; Gold et al. 2000), and enhancing pulmonary and systemic inflammation (Ghio et al. 2000; Peters et al. 2001).

Arterial stiffness with increased blood pressure may be in the mechanistic pathway linking air pollution to cardiovascular risk. Arterial stiffness refers to the artery’s capacity to expand and contract in response to cardiac flow (Arnett et al. 1994). The underlying physiological mechanisms for stiffening of arteries are both functional and structural (Glasser et al. 1997). Functional determinants include “neurohumoral influences, such as the renin-angiotensin system, the adrenergic nervous system, and endothelium-derived factors” (Glasser et al. 1997). Structurally, elastin in the vessel wall may thin and fracture because of aging and such conditions as hypertension and increased collagen deposition can reduce compliance of the vessel wall (Glasser et al. 1997). Other structural influences include smooth muscle tone or smooth muscle cell hypertrophy and cell–cell and cell–matrix attachments (Glasser et al. 1997).

Stiffer arteries are linked with higher pulse pressure and adverse ventricular remodeling (Arnett et al. 1994; O’Rourke and Mancia 1999; Rowe 1987), which along with hypertension are major risk factors for cardiovascular outcomes (Malhotra et al. 2003; Meaume et al. 2001). Therefore, various measures of arterial stiffness predict risk of future cardiovascular events (Arnett et al. 1994; Liao et al. 1999; Pannier et al. 2005) and are correlated with other known cardiovascular risk factors (Salomaa et al. 1995).

Arterial stiffness has been related to exposure to tobacco smoke (Tanaka and Safar 2005), but previous findings have not been uniformly consistent (Din-Dzietham et al. 2000; Kool et al. 1993; Levenson et al. 1987; Liang et al. 2001; Mack et al. 2003; Mahmud and Feely 2003; Mitchell et al. 2007), and results differed according to which measure of arterial stiffness was examined (Li et al. 2005). Although changes in blood pressure and vasoconstriction have been associated with short-term exposure to outdoor air pollution (Auchincloss et al. 2008; Brook et al. 2002; Zanobetti et al. 2004), the relationship between long-term exposure to ambient pollution and the development of arterial stiffness has not been studied.

We hypothesized that long-term (20-year) exposure to the mass concentrations of airborne particles ≤ 2.5 and 10 μm in aerodynamic diameter (PM_2.5_ and PM_10_, respectively)would be positively associated with three measures of current arterial stiffness in adulthood among participants of the Multi-Ethnic Study of Atherosclerosis (MESA).

## Materials and Methods

### Study population

Study participants were a subset of the 6,814 men and women 44–84 years of age enrolled in MESA, an ongoing longitudinal study of subclinical atherosclerosis funded by the National Heart, Lung and Blood Institute (Bild et al. 2002). Participants were free of clinical cardiovascular disease at enrollment and were recruited from six U.S. field centers: Baltimore, Maryland (Johns Hopkins University); Chicago, Illinois (Northwestern University); Winston-Salem, North Carolina (Wake Forest University); Los Angeles, California (University of California–Los Angeles); New York, New York (Columbia University); and St. Paul, Minnesota (University of Minnesota), as described in detail elsewhere (Bild et al. 2002). The present analyses used data collected at the baseline visit (June 2000–August 2002). The study was approved by the relevant institutional review committees, and participants gave informed consent.

### Arterial stiffness outcomes

Two noninvasive measurement technologies were used on all MESA participants during the baseline physical exam: radial arterial applanation tonometry and carotid artery ultrasound. These techniques and the parameters calculated from them have been validated and used in previous studies (Cohn et al. 1995; Gamble et al. 1994).

Arterial applanation tonometry is used to assess the form of the pulse wave measured at the radial artery (Cohn et al. 1995). The waveform, which changes with aging and various disease states, is from the pressure wave created by the left ventricle’s contraction and the second wave reflected back once it reaches the small arteries (Duprez et al. 2004). From the waveform, using a theoretical model of the vasculature [modified Windkessel model (O’Rourke et al. 2002)], indices of large artery (capacitative) compliance (C_1_) (mL/mmHgv × 10) and small artery (oscillatory) compliance (C_2_) (mL/mmHg × 100) are calculated by extracting the decay component of the waveforms and correcting for systemic vascular resistance, age, sex, and estimated body surface area (Tao et al. 2004).

During the exam, MESA participants rested for at least 15 min lying down, and after the wrist was stabilized, a sensor (tonometer) was placed over the radial artery for 20 sec. The identical protocol and equipment (CVProfilor DO-2020 CardioVascular System; Hypertension Diagnostics, Inc., Eagan, MN, USA) were used at all six centers, and blood pressures were taken at the same time as the pulse waveform. The waveform tracings were averaged into a representative waveform, and C_1_ and C_2_ were calculated. Lower values of C_1_ and C_2_ indicate greater arterial stiffness (less compliance). C_1_ and C_2_ estimate the systemic arterial compliance. C_1_ is more dependent on pressure than is C_2_, so reduced C_2_ may reflect a change in the structure or function of the vessel wall and may be an earlier marker of disease (Cohn et al. 2004; Duprez et al. 2004).

Carotid ultrasound is used to capture images of the common carotid artery that are used to calculate parameters representing the mechanical behavior of the wall of this elastic artery. These parameters are intended to reflect the artery’s ability to expand in response to pulse pressure. Changes in these mechanics probably represent alterations in the arterial wall. Reduced elasticity of the carotid artery has been associated with cardiovascular outcomes and risk factors in population-based studies (Liao et al. 1999; Salomaa et al. 1995).

Carotid ultrasound was performed on MESA participants using the same protocol in all six sites after they had rested 5 min in a quiet semidarkened room. A B-mode ultrasound machine (Riley et al. 1992) (Logiq 700 ultrasound machine; General Electric Medical Systems, Milwaukee, WI, USA) was used to capture images of the common carotid artery by certified sonographers. The ultrasound technician placed the transducer on the participant’s neck, approximately 1 cm below the carotid bulb, in order to capture the images (Riley et al. 1992). Images were taken for 30 sec, and blood pressure measurements were made by upper arm automated sphygmomanometer (brachial artery) before and after the ultrasound image acquisition. The digitized carotid arterial diameter data from the ultrasound were then read (in replicate by different readers to estimate variability) to obtain average diastolic and systolic diameters from as many as ten cardiac cycles. The wall thickness measurements were calculated from the B-mode images, representing the combined thickness of the intima, media, and adventitia (Gamble et al. 1994). Readings were performed by readers with no information on the patients or their pollution exposure. From these measurements and from the mean brachial artery systolic and diastolic measurements made before and after the ultrasound, Young’s modulus (YM) was calculated (Gamble et al. 1994). The YM is a measure of elasticity, adjusted for wall thickness, and is defined as “the pressure step per square centimeter required for (theoretical) 100% stretch from resting length” (O’Rourke et al. 2002) and calculated using systolic and diastolic blood pressure (SBP and DBP, respectively) and the carotid artery diameters from the ultrasound data: Δ (SBP − DBP) × diastolic diameter ÷ (Δ systolic − diastolic diameter × wall thickness), expressed as millimeters of mercury per centimeter (O’Rourke et al. 2002). Higher values signify greater arterial stiffness. In this sample, YM and another measure of carotid artery distensibility, the distensibility coefficient (Gamble et al. 1994), were highly negatively correlated (for the log-transformed versions: Pearson correlation coefficient = −0.82, *p* < 0.0001), so we chose to use only the YM metric.

Increased arterial stiffness is indicated by an increase in YM and decreases in C_1_ and C_2_. These measures provide complementary information in that YM reflects properties of the elastic carotid artery and is calculated based on a specific measure of diameter and pressure. Rather than being direct measures, C_1_ and C_2_ are intended to reflect properties of the entire pools of large and small arteries and are calculated using the modified Windkessel model of the vasculature (O’Rourke et al. 2002). YM had low correlations with C_1_ (−0.15) and C_2_ (−0.20).

### Air pollution exposure

Average long-term exposure to particle mass for MESA participants was estimated using three metrics that were calculated using the complete residential history that participants provided during the two decades preceding the baseline clinical exam (2000–2002). We investigated a 20-year exposure because arterial stiffness may develop progressively over long periods, and 20 years was the time period of residential history available for the MESA participants. Because different measures may have different degrees of measurement error, we contrasted three metrics of long-term exposure: observed PM_10_ from monitors nearest participants’ residences, imputed PM_10_ derived from a space–time model, and imputed PM_2.5_ also derived from a space–time model. Observed PM_10_ was derived from community monitors sited for regulatory purposes from the U.S. Environmental Protection Agency (EPA)Aerometric Information Retrieval Service (AIRS) database (U.S. EPA 2003). Observed PM_2.5_ could not be estimated because PM_2.5_ has been systematically monitored only in recent years. Particle monitors collected 24-hr integrated samples, some daily but most every third day. Participants reported residential locations for each month between January 1982 and the date of the baseline exam, including move dates (month and year), and average monthly exposures were assigned using the nearest monitors with available data on the days within each month. The mean distance to the closest monitor was 9 km (range, 0.45–51 km); because 51 km is a relatively large distance, exposure misclassification may be greater for those participants who lived farthest from the monitors. An area-under-the-curve (AUC) measure was computed by numerical integration of each monthly average of the directly monitored PM_10_ values.

The imputed cumulative PM_2.5_ and PM_10_ exposures were derived from a space–time model using monitored PM; temperature and airport visibility data from the National Climatic Data Center (National Oceanic and Atmospheric Administration 2003); total suspended particle and carbon monoxide, sulfur dioxide, nitrogen dioxide, and ozone levels from the AIRS network; and population density from the 1990 U.S. Census (Raghunathan et al. 2006). Spatial effects were modeled with thin plate splines, and time effects were modeled with trend, cyclic, and autoregressive terms. Monthly PM exposure was multiply imputed (40 imputations) for each month and each location represented in the residential history data using the nationwide exposure surface created from the space–time model. These data sets, created using multiple imputation, were combined for single cumulative imputed 20-year exposures to PM_2.5_ and PM_10_ for each participant, represented by an AUC. Further details are provided elsewhere (Diez-Roux et al. 2008). Multiple imputation was performed using the space–time model to improve the validity of the estimates (Raghunathan et al. 2006).

To facilitate comparisons with other air pollution studies published with MESA data (Diez-Roux et al. 2008), we also investigated associations of the outcomes with the mean annual concentrations of PM_10_ and PM_2.5_ during 2001, using the closest monitor. In sensitivity analyses, associations of long-term exposures were also investigated after adjustment for recent exposures. This was accomplished by taking the difference between both the mean PM_2.5_ and PM_10_ concentrations for the week before the baseline examination, based on the closest population-based monitor to the residential address, and the long-term (20-year) exposures for each participant and including that difference value as a term in the model. The coefficient for the new difference variable represents the short-term exposure effects, and the coefficient for the long-term exposure represents the long-term exposure effect, controlling for short-term exposure.

### Other covariates

At the MESA baseline exam, height, weight, and waist and hip circumference were measured using standard procedures. Resting seated blood pressure was measured with an automated oscillometric sphygmomanometer (Dinamap PRO 100; Critikon, Tampa, FL, USA). Mean arterial pressure (MAP) was calculated from SBP and DBP as [(2 × DBP) + SBP] ÷ 3. Fasting blood glucose and triglycerides were also measured with standard methods. Diabetes was defined as fasting blood glucose of ≥ 126 mg/dL or use of a diabetes medication (insulin or oral hypoglycemic agents), and impaired glucose tolerance as fasting blood glucose between 100 and 125 mg/dL. Participants provided detailed information on personal characteristics: sex, age, race/ethnicity, cigarette-smoking status at the day of the examination (never, former, current), pack-years and cigarettes per day if ever smoked, and diabetes status. They were also asked to report on exposure (hours per week) to environmental tobacco smoke (ETS) in the year before the examination (i.e., being in “close quarters” with a person who smoked at home, at work, in a car). Physical activity was reported as total of light, moderate, and vigorous activity per week.

### Statistical approach

In the initial analyses, we calculated correlations between air pollutant metrics; after examining distributional statistics, all three arterial stiffness outcome variables were log-transformed for the analysis.

We chose several covariates *a priori* based on previously reported associations with arterial stiffness and first-fit linear regressions that assessed all covariate relations with the outcome variables, not including the air pollutant metrics. We then used random effects regression models (with a random intercept for each site) to examine associations between our three air pollution exposures (observed PM_10_, imputed PM_10_, and imputed PM_2.5_) and three arterial stiffness outcome variables (YM, C_1_, and C_2_) in three progressive steps. The first model included the air pollution exposure and weather and season (see below) with no other covariates; the second adjusted for age, sex, and race; and the third included all additional covariates [fasting blood glucose, triglycerides, diabetes, MAP, cigarette-smoking status, pack-years, cigarettes per day (for current smokers), ETS, waist:hip ratio, and physical activity]. MAP was included because it had a more consistent association with arterial stiffness in a literature review than did other blood pressure parameters. Models examining C_1_ and C_2_ were also adjusted for heart rate, weight, and height, because these variables are used in the algorithm to calculate the C_1_ and C_2_ parameters, and adjusting for them allows isolation of the arterial compliance effect.

Because of the differing climate regions in which MESA sites are found, control for weather and season was accomplished following an approach previously applied in this cohort (Park et al. 2010). We included interaction terms between site and splines with 3 degrees of freedom for apparent temperature (a construct of dew point and temperature that better reflects the physiological experience of weather) to the models, because the shape of the spline could have differed according to site. To remove any further residual confounding by season, we included indicator variables to represent months with average monthly temperatures above 10°C (50°F) during the baseline recruitment period (all year for Los Angeles; May to October for Chicago and St. Paul; and April to October for the remaining three sites).

We fit regression models pooled across sites with a random effect for study site because *a*) there were site-specific quantitative differences in the mean and range of our outcome measures and possibly within-site correlations in outcomes due to unmeasured site-level factors; *b*) pollutant concentrations, mix, and composition can differ greatly by location, and we used a particle-mass metric and not composition data; and *c*) there was some evidence of heterogeneity by site (*p*-values for site by pollution interactions were 0.04, 0.05, and 0.22 for YM, C_1_, and C_2_, respectively). The fixed effect coefficient for the PM exposure was used to estimate associations of PM with the outcomes pooled across the six study sites. However, in sensitivity analyses, we also examined site-specific results because of the possibility of heterogeneity across sites in the associations (because of differential particle composition or other sources of heterogeneous effects), although these analyses are limited by sample size and by reduced variability in exposures within sites.

To assess nonlinearity of the association between pollution and the three outcomes, we fit generalized additive models to site-stratified data and plotted adjusted associations by each of the six MESA sites. No evidence for nonlinearity was found, so linear terms for the pollution variables were used in all models. We followed a similar approach to model the association with continuous age and pack-years (with pooled data from all sites), but there was no evidence for a nonlinear effect of age or pack-years, so linear terms for these variables were retained in the models.

We examined potential effect modification by body mass index (BMI), waist:hip ratio, diabetes, physical activity, age, sex, and race/ethnicity in fully adjusted models by including the corresponding multiplicative interaction term(s). Important differences in the process of aging and the development of arterial stiffness have been noted by sex (Smulyan et al. 2001), and associations between air pollution and heart rate variability have differed by race and ethnicity (Liao et al. 2004). The other variables have been markers of differential vulnerability to air pollution in other studies. We defined effect modification as present if the *p*-value for the interaction term was < 0.05. Because of the importance of tobacco-smoke exposure, we also stratified the population according to smoking status (never, former, and current), as well as among nonsmokers not exposed to second-hand smoke (interaction terms or stratified), and fit fully adjusted random effects models to these subsets. We also conducted a sensitivity analysis restricting the models to participants for whom the nearest monitor was within 10 km.

Regression results are reported as percent differences in the outcome variables at the baseline exam [100 × (exponentiated mean difference − 1)] associated with a 10-μg/m^3^ increase in particle exposure. We performed the analyses using the SAS statistical package (version 9.2.1; SAS Institute, Inc., Cary, NC). Statistical significance was defined as *p* < 0.05.

Of the 6,814 participants enrolled in MESA, 5,286 had complete data on the outcome and clinical covariates used for the analyses and had completed the single residential history questionnaire covering exposure during 1982–2002. Of these, 4,570 had latitudes and longitudes available for all residential addresses between August 1982 and August 2002 and thus complete data on the average long-term exposures. Of these, three were excluded because of reporting pack-years of smoking greater than 200. Restricting to participants who had data for weather and season yielded a total of 3,996 participants for analysis with complete information on all covariates and all outcomes.

## Results

Table 1 shows the demographic and clinical characteristics of the sample. The average age of participants in the study sample was 62 years, and a little more than half were women. About half the participants had ever smoked, and about 36% reported any ETS exposure. No important differences in key characteristics existed between the analysis sample and the full MESA cohort (*n* = 6,814; data not shown). The study sample had fewer Chinese and Hispanic participants than did the full cohort (which had 11.8% and 22% of these ethnicities) because many in these groups were recent immigrants whose average long-term exposure could not be estimated. Correlations among all three arterial stiffness variables were −0.26 and −0.17 between YM and C_1_ and C_2_, respectively, and 0.48 between C_1_ and C_2_ (all *p* < 0.0001). Clinical measures were consistent with expectation.

Table 2 shows the pollution levels and outcome variables by site. Los Angeles had the highest mean pollution levels, and Winston-Salem and St. Paul had the lowest. For the pollution levels estimated in the two decades before the MESA baseline exam, the imputed and directly observed PM_10_ levels are comparable, with the imputed levels slightly lower on average than the observed. Measures of arterial stiffness differed by study site: They were most unfavorable (higher for YM, lower for C_1_ and C_2_) in New York City (YM), Baltimore (C_1_), and Winston-Salem (C_2_).

In the multivariable linear regression models that did not include the pollutant values (Table 3), age was associated with stiffer arteries, and women had stiffer arteries than did men for all three measures of stiffness examined. Chinese, Hispanic, and African American participants tended to have stiffer arteries than did Caucasians based on YM and C_2_, but we observed the opposite for C_1_, although differences were sometimes not statistically significant. YM was lower in former and current smokers than in never smokers, which indicated less stiff arteries. However, current smoking was associated with greater arterial stiffness as assessed by C_2_. Smoking status was not associated with stiffness as assessed by C_1_. Exposure to ETS and smoking exposure represented by pack-years were not associated with increased arterial stiffness for any of the outcome variables. Associations of smoking status and pack-years with the outcomes were generally similar when both variables were not simultaneously in the same model. Greater MAP was associated with greater stiffness by all three measures. Physical activity was not significantly associated with any of the outcomes, nor was diabetes status, glucose, or triglycerides.

In all of the models, including those adjusted for all covariates (for which none of the variance inflation factors exceeded 3), average long-term exposure to PM_10_ was not associated with greater arterial stiffness measured by YM (Table 4). The same was true for the compliance outcomes C_1_ and C_2_ at all sites. The 2001 nearest-monitor average PM_10_ and PM_2.5_ were also not associated with the outcomes for any of the sites.

We then took study site into consideration. Patterns of association calculated for the six sites in site-stratified models were generally consistent with no associations for all pollution metrics and covariate adjustment schemes (Tables 5,6). Although a scattering of associations were significant, they were not robust to the covariate adjustments for all the models.

Analyses with adjustment for shorter term PM exposure did not alter the patterns in the observed associations (data not shown). Analyses restricted to participants residing within 10 km of the nearest monitor (*n* = 2,518) were consistent with the analysis of the complete data set (data not shown).

We examined heterogeneity by BMI, waist:hip ratio, diabetes, physical activity, sex, race/ethnicity, and age in fully adjusted models. Race had significant interaction terms for YM and C_1_ and diabetes for C_1_ and C_2_. None of the other variables showed evidence of effect modification.

We fitted fully adjusted random effects models stratified by race and diabetes for the outcomes for which interaction terms were significant. Among black participants, the percent difference in YM with a 10-μg/m^3^ increase in nearest-monitor PM_10_ was 14.8% [95% confidence interval (CI), 1.3–30.2%] and with a 10-μg/m^3^ increase in imputed PM_10_ was 16.9% (95% CI, 1.9–34.1%). CIs for corresponding estimates for the other race/ethnic groups did not include the point estimates for blacks for these metrics, except for Chinese participants and nearest-monitor PM_10_ (1.2%; 95% CI, −10.9% to 15.0%). We observed no significant associations for YM in other race/ethnic groups; in general, the point estimates of effect were lowest for Hispanics for YM for all three metrics. For C_1_, white participants had a significant positive association with imputed PM_2.5_ (4.4%; 95% CI, 0.2–8.9%), and Chinese participants, for imputed PM_10_ (13.5%; 95% CI, 3.5–24.4%). We observed no other consistent patterns of point estimates by race/ethnicity. For diabetes-stratified analyses, patterns of association were inconsistent when comparing pollution metrics and outcomes, with two positive and one negative significant association among those with no diabetes and those with impaired glucose tolerance, respectively.

For the results stratified by smoking status, we found only two significant associations. Among the 1,519 former smokers, C_1_ was associated with both observed −8.9% (95% CI, −16.4% to −0.7%) and imputed −8.9% (95% CI, −16.8% to −0.4%) PM_10_. No other associations were significantly different from zero, and point estimates showed no consistent trends by smoking status.

## Discussion

Higher estimated long-term particulate mass exposure was not consistently associated with greater arterial stiffness among MESA participants. For the few arterial stiffness outcomes that showed associations with air pollution exposure when examining subgroups by race, diabetes, or smoking status, we found no consistent pattern across all three particle exposure metrics or adjustment schemes, suggesting that chance may explain these findings. For the pulse-wave outcomes, increased long-term exposure to PM was not associated consistently or significantly with lower arterial compliance as represented by C_1_ (large artery compliance) or C_2_ (small artery compliance). These results do not support our hypothesis that long-term PM mass exposure is associated with greater stiffness.

A strength of this study was the simultaneous use of multiple subclinical markers of arterial stiffness. These measures could be used in other studies that examine risk factors for arterial stiffness. Several different indices, derived from noninvasive physiological measurements, and referred to as compliance, distensibility, and elasticity, have been used to characterize different aspects of stiffness in large and small arteries, as discussed in recent reviews (Oliver and Webb 2003; O’Rourke and Mancia 1999; O’Rourke et al. 2002). Ultrasound-assessed arterial distensibility measures the relative diameter change in a vessel for a given pressure increment. Indirect stiffness indicators include pulse-wave–derived measures such as large- and small-artery elasticity indices as well as systemic vascular resistance. Using several measures in arteries of differing structures and sizes can provide an overall sense of arterial characteristics in any given individual (O’Rourke and Mancia 1999).

Tobacco smoke exposure is an important analogy to ambient PM, given similarities between tobacco smoke and ambient particle pollution (Hinds 1978). In this cohort, former and current smoking status was associated with reduced arterial stiffness as represented by YM, consistent with a previous analysis in MESA participants (Sharrett et al. 2006), but current smoking was associated with more arterial stiffness as measured by C_2_. Pack-years of smoking and ETS exposure were weakly associated with greater stiffness, but associations were statistically significant only for C_2_. Previous studies (Din-Dzietham et al. 2000; Kool et al. 1993; Levenson et al. 1987; Li et al. 2005; Liang et al. 2001; Mack et al. 2003; Mahmud and Feely 2003; Mitchell et al. 2007; Simons et al. 1998; Stefanadis et al. 1998) have shown conflicting results with respect to the association between smoking and arterial stiffness. Notably, self-reported ETS exposure was linked with increased arterial stiffness, in a dose-dependent manner, although only among people with high BMI and increased carotid intima-media thickness, in a cross-sectional study of 227 healthy, nonsmoking adults from the Vitamin E Atherosclerosis Prevention Study (Mack et al. 2003). In other studies, long-term smoking behavior in 185 younger, otherwise healthy subjects was also associated with greater arterial stiffness, as measured by increased aortic SBP and augmentation index and reduced aortic-brachial pulse pressure amplification (Mahmud and Feely 2003). Among 145 nonsmokers and 142 smokers from the Bogalusa Heart Study (mean age, 36 years), smoking was associated with impaired small artery compliance and increased systemic vascular resistance as determined from waveforms taken in the radial artery (Li et al. 2005). Other research in a population of generally healthy adults 45–64 years of age from the Atherosclerosis Risk in Communities cohort showed no association with pulsatile arterial diameter change in the carotid artery (Din-Dzietham et al. 2000). Explanations offered for the differing results include the variety of outcome measures used, study designs varying from controlled exposures to tobacco smoke to reported smoking behavior in epidemiological cohorts, and the complexity of determinants of arterial stiffness (elastin, collagen, smooth muscle) and how these functional and structural aspects of the arteries may be affected by tobacco and air pollution exposure. Future work in the MESA cohort will examine associations of smoking with arterial stiffness using more sophisticated exposure measures such as cotinine levels and may shed light on the internally contradictory associations within MESA.

C_1_ can be said to reflect compliance in the larger arteries, and C_2_ compliance, of the smaller arteries [and therefore may provide some information on endothelial function, because their compliance depends less on elasticity and more on smooth muscle function, regulated by the endothelium (Tao et al. 2004)], although evidence for C_2_ being a reliable indicator of endothelial function is mixed (Westhoff et al. 2007). C_1_ and C_2_ measure general arterial stiffness throughout the respective arterial pools and in this way differ from YM in the carotid artery, which is specific to a particular arterial location. Because the carotid is a large artery, it may not reflect small arterial stiffness. Further, stiffness is not directly measurable in any particular small artery. The specific vascular measures we used are not the only ones available; for example, central measures (aortic) could have different associations.

Error in assigning environmental exposure to participants is probable in this study and could be seen as a limitation. Indeed, one of the primary limitations is that we assessed exposure using PM mass alone and did not estimate associations with the constituent components of the mass that may be most related to the health effects. Ambient PM tends to be a spatially homogeneous pollutant (Salmon et al. 1999), and background exposures are likely to be fairly well represented by ambient monitor measures (Sarnat et al. 2005), especially among MESA participants who reported spending 60% of their time at home or within 2 km of their homes in the period before the baseline exam (Diez-Roux et al. 2006). However, the components and types of ambient particles (fine particles, traffic-associated particles) that may be most relevant to cardiovascular health are probably better measured at a smaller spatial scale. Planned future work in the MESA cohort will use time–activity diaries and additional fixed and person-level monitoring to enhance exposure assessment, including evaluating particle components and the relevance of different size fractions, and average long-term exposure modeling using distance to roadways and traffic counts may help us ascertain the validity of the findings presented here. The lack of clear, consistent effects of air pollution on the vascular outcomes we assessed in these MESA participants could be related to the fact that they were free of clinical cardiovascular disease when enrolled, but also could relate to limitations in the exposure metrics we used.

## Conclusions

We evaluated long-term particle mass exposure in relation to three measures of arterial stiffness measured at one point in time in a large, multiethnic sample with a substantial age range and extensive information on covariates. None of the measures was consistently associated with air pollution in this sample. This single study’s results do not rule out a relationship between ambient air pollution exposure and chronic vascular changes. Indeed, the relatively narrow inter- and intracity gradients in exposure and limitations in estimating these exposures, among other factors, may have contributed to the null findings.

Further exploration, in both epidemiological and toxicological studies, is needed regarding relations between higher pollution exposures and arterial stiffness outcomes. Exposure assessments that provide a more complete picture of both the PM size fractions and components (e.g., metals and organics) relevant to cardiovascular health will also be helpful and will form part of an ancillary study of the MESA cohort. Analyses using both ultrasound and pulse-wave–derived indices within the same populations, of similar high quality as those we had available in MESA, and evaluating repeated measures of arterial stiffness within the same participants will be useful for understanding the full complexity of these relations.

## Figures and Tables

**Table 1:** Demographic and clinical characteristics and tobacco smoke exposure for participants included in the analyses (*n* = 3,996): MESA, 2000–2002.

Characteristic	Mean ± SD or percent
Demographic characteristics	
Age (years)	61.84 ± 10.01
Women (%)	52.1
Race/ethnicity (% distribution)	
Caucasian	43
Chinese	7.7
African American	27.9
Hispanic	21.4
Study site (% distribution)	
Baltimore	14
Chicago	18.8
Winston-Salem	18
Los Angeles	17.1
New York City	14.4
St. Paul	17.6

Tobacco smoke exposure	
Smoking status (% distribution)	
Never	49.5
Former	38
Current	12.5
ETS exposure^a^ (% distribution)	
None	63.4
≥ 1 hr/week	36.6
Pack-years of cigarette (packs/day × year)	11.4 ± 20.7
Cigarettes/day^b^	1.6 ± 5.7
Reported total physical activity^c^	
Low	25.6
Medium	50.2
High	24.2

Clinical characteristics	
Waist:hip ratio	0.93 ± 0.08
Weight (kg)	79.7 ± 16.9
Height (cm)	167.0 ± 10.0
Heart rate (beat/min)	62.9 ± 9.6
Fasting glucose (mg/dL)	103.3 ± 28.2
Triglycerides (mg/dL)	131.9 ± 86.4
MAP (mmHg)	95.8 ± 13.3
Diabetes (% distribution)	
None	59.6
Impaired glucose (mg/dL)	27.3
Diabetes	13.1

a Asked only of never or former smokers.

b Asked only of current smokers.

c Low, 9.1 hr/day; medium, 9.1–15.5 hr/day; high, > 15.5 hr/day.

**Table 2:** Long-term PM exposure and outcomes (mean ± SD) averaged across all sites and by individual site for participants included in the analyses (*n* = 3,996): MESA, 2000–2002.

Outcome	Pooled	Winston-Salem	New York City	Baltimore	St. Paul	Chicago	Los Angeles
Arterial stiffness outcomes	*n* = 3,996	*n* = 720	*n* = 575	*n* = 560	*n* = 705	*n* = 753	*n* = 683
YM (mmHg/cm)	1277.5 ± 637.7	1221.5 ± 643.82	1364.9 ± 748.89	1263.9 ± 619.93	1197.8 ± 430.23	1341.4 ± 538.37	1285.9 ± 791.34
C_2_ (mL/mmHg × 10)	13.51 ± 5.44	13.63 ± 5.48	13.48 ± 4.96	12.30 ± 5.02	13.71 ± 5.06	14.47 ± 6.00	13.16 ± 5.61
C_1_ (mL/mmHg × 100)	4.54 ± 2.80	4.14 ± 2.52	4.37 ± 2.68	4.37 ± 2.58	5.55 ± 3.08	4.57 ± 2.92	4.17 ± 2.68
PM exposures in preceding 20 years (average)							
Observed PM_10_^a^	34.21 ± 7.07	28.43 ± 1.90	31.52 ± 3.10	32.82 ± 1.31	29.41 ± 2.60	35.00 ± 2.57	47.78 ± 3.65
Imputed PM_10_^b^	33.84 ± 7.10	28.56 ± 1.74	32.28 ± 2.04	31.82 ± 1.41	27.17 ± 2.39	35.79 ± 2.78	47.10 ± 3.43
Imputed PM_2.5_^b^	21.47 ± 5.00	19.04 ± 2.58	21.67 ± 2.86	22.56 ± 2.52	15.12 ± 2.84	23.82 ± 3.29	26.94 ± 4.72
PM exposures in 2001 (yearly average)^c^							
PM_10_	29.73 ± 7.63	22.87 ± 1.16	26.34 ± 7.88	25.72 ± 2.45	28.95 ± 2.60	30.38 ± 3.57	43.20 ± 2.48
PM_2.5_	16.80 ± 3.90	15.25 ± 0.68	15.73 ± 1.11	15.66 ± 0.95	12.81 ± 0.75	17.06 ± 1.01	24.09 ± 3.28

a Average AUC of observed PM_10_ from nearest monitor for previous 20 years.

b Mean of 40 imputed values from space–time model.

c Yearly average PM from nearest monitor during 2001.

**Table 3:** Percent difference (95% CI) in three arterial stiffness outcomes, not adjusted for pollution (*n* = 3,996): MESA, 2000–2002.

Covariate	YM	C_1_	C_2_
Age (10 years)	7.1 (5.6 to 8.7)	−11.3 (−12.2 to −10.4)	−16.5 (−17.8 to −15.2)
Males	−11.1 (−13.6 to −8.6)	11.4 (8.5 to 14.3)	20.9 (16.0 to 25.9)
Race/ethnicity			
Caucasian	Reference	Reference	Reference
Chinese	9.7 (3.9 to 15.8)	7.7 (3.7 to 11.9)	−4.3 (−9.8 to 1.4)
African American	3.2 (−0.2 to 6.7)	4.9 (2.6 to 7.2)	−8.9 (−12.0 to −5.7)
Hispanic	6.7 (2.8 to 10.6)	2.1 (−0.5 to 4.8)	−4.2 (−7.9 to −0.2)
Glucose (mg/dL)	0.3 (−1.0 to 1.5)	0.6 (−0.2 to 1.5)	−1.3 (−2.5 to 0.0)
Triglycerides/10 (mg/dL)	2.0 (−1.0 to 5.0)	−0.6 (−2.5 to 1.4)	−1.9 (−4.7 to 1.1)
Diabetes			
Never	2.0 (−1.2 to 5.4)	−2.1 (−4.2 to 0.0)	−1.2 (−4.4 to 2.2)
Impaired glucose (mg/dL)	−1.3 (−7.0 to 4.7)	−6.7 (−10.2 to −3.0)	2.1 (−3.9 to 8.5)
Diabetes	Reference	Reference	Reference
MAP/10	6.7 (5.6 to 7.8)	−11.2 (−11.8 to −10.6)	−15.0 (−15.9 to −14.1)
Smoking status			
Never	Reference	Reference	Reference
Former	−3.7 (−6.8 to −0.5)	1.4 (−0.7 to 3.7)	−1.0 (−4.3 to 2.4)
Current	−13.1 (−18.4 to −7.5)	−1.7 (−5.6 to 2.4)	−12.7 (−18.1 to −7.0)
Pack-years/10	0.3 (−0.6 to 1.1)	−0.3 (−0.8 to 0.3)	−1.3 (−2.2 to −0.5)
Cigarettes/day/10	0.5 (−3.3 to 4.4)	0.0 (−2.5 to 2.5)	−1.6 (−5.3 to 2.3)
Any ETS exposure	1.5 (−1.4 to 4.4)	0.0 (−1.8 to 1.9)	−2.9 (−5.7 to 0.0)
Waist:hip ratio	24.9 (3.9 to 50.1)	5.4 (−7.6 to 20.2)	−30.5 (−43.4 to −14.8)
Physical activity^a^			
Low	Reference	Reference	Reference
Medium	0.8 (−2.5 to 4.1)	−1.3 (−3.4 to 0.9)	1.7 (−1.6 to 5.2)
High	0.4 (−3.4 to 4.4)	−3.1 (−5.5 to −0.6)	0.4 (−3.5 to 4.5)
Heart rate/10^b^ (beats/min)		−8.5 (−9.4 to −7.7)	−0.7 (−2.1 to 0.7)
Weight/10^b^ (kg)		1.4 (1.0 to 1.7)	4.0 (3.5 to 4.5)
Height/10^b^ (cm)		9.9 (8.3 to 11.5)	8.9 (6.4 to 11.3)

Regression results reported as percent difference (95% CI) in the outcome variables [100 × (exponentiated mean difference − 1)]. Positive association with YM indicates greater arterial stiffness; negative association with C_1_ and C_2_ indicates reduced compliance; associations are adjusted for all other variables in the table.

a Physical activity was based on total min/day: low represents lowest quartile of reported activity, medium refers to the 25–75th percentiles, and high was the highest quartile.

b Heart rate, weight, and height were adjusted only for outcomes C_1_ and C_2_; this adjustment allowed the isolation of the arterial compliance effect.

**Table 4:** Percent difference (95% CI) in three arterial stiffness outcomes per 10-μg/m^3^ PM increment in preceding two decades and 2001 PM exposure under various covariate adjustment schemes: MESA, 2000–2002.

	PM exposure in preceding two decades			2001 PM exposure	
Outcome/covariates	Observed PM_10_	Imputed PM_10_	Imputed PM_2.5_	PM_10_	PM_2.5_
YM					
PM (weather/season only)^a^	3.5 (−2.5 to 9.8)	5.2 (−0.3 to 11.0)	0.4 (−3.6 to 4.6)	−0.2 (−0.8 to 0.4)	−0.8 (−1.4 to −0.3)
Add age, sex, race	2.3 (−3.5 to 8.6)	3.2 (−2.4 to 9.1)	−0.8 (−4.7 to 3.2)	0.0 (−0.5 to 0.5)	−0.5 (−1.1 to 0.0)
All covariates^b^	1.6 (−3.8 to 7.3)	3.1 (−1.9 to 8.3)	−1.4 (−5.1 to 2.4)	0.0 (−0.5 to 0.5)	−0.5 (−1.1 to 0.0)

C_1_					
PM (weather/season only)^a^	2.7 (−2.4 to 8.1)	0.7 (−5.0 to 6.7)	−0.2 (−3.0 to 2.6)	−0.2 (−0.6 to 0.3)	−0.6 (−1.5 to 0.2)
Add age, sex, race	2.9 (−1.6 to 7.7)	2.6 (−2.2 to 7.7)	1.7 (−0.9 to 4.3)	−0.2 (−0.5 to 0.2)	0.0 (−0.8 to 0.8)
All covariates^b^	2.9 (−1.8 to 7.7)	2.6 (−2.3 to 7.8)	1.5 (−1.0 to 4.1)	−0.2 (−0.5 to 0.2)	0.0 (−0.8 to 0.8)

C_2_					
PM (weather/season only)^a^	−1.8 (−6.9 to 3.5)	−3.9 (−9.6 to 2.1)	−3.7 (−8.0 to 0.8)	0.3 (−0.3 to 0.8)	0.6 (−0.2 to 1.4)
Add age, sex, race	−3.3 (−8.0 to 1.6)	−1.4 (−6.5 to 3.9)	−0.7 (−4.6 to 3.3)	0.1 (−0.4 to 0.6)	0.2 (−0.6 to 1.0)
All covariates^b^	−3.6 (−8.2 to 1.3)	−1.5 (−6.5 to 3.8)	−1.2 (−5.0 to 2.7)	0.1 (−0.3 to 0.5)	0.2 (−0.6 to 1.0)

Regression results are reported as percent differences (95% CIs) in outcome variables [100 × (exponentiated mean difference − 1)] for each metric and outcome, pooled over all study sites using random effects regression. A positive point estimate for YM indicates greater arterial stiffness with higher exposure; a negative point estimate for C_1_ and C_2_ indicates reduced compliance with higher exposure. PM levels are from the nearest monitor or space–time model (see Table 2).

a Weather and season terms included in all models: apparent temperate spline with 3 degrees of freedom, interacted with site; and indicator variables to represent months with average monthly temperature above 10°C (50°F) during baseline recruitment period (all year for Los Angeles; May–October for Chicago and St. Paul; April–October for remaining three sites).

b All covariates for YM are age, sex, race, fasting blood glucose, triglycerides, diabetes, seated MAP, smoking status, pack-years, cigarettes per day, ETS exposure, physical activities, waist:hip ratio, season, and weather. For C1 and C2, in addition, height, weight, and heart rate were included.

**Table 5:** Percent difference (95% CI) in three arterial stiffness outcomes per 10-μg/m^3^ increment in long-term PM exposure under various covariate adjustment schemes, stratified by study site: MESA, 2000–2002.

Outcome and covariates	YM			C_1_			C_2_		
	PM_10_		Imputed PM_2.5_	PM_10_		Imputed PM_2.5_	PM_10_		Imputed PM_2.5_
	Observed	Imputed		Observed	Imputed		Observed	Imputed	
New York (*n* = 575)									
PM (weather/season only)^a^	−3.4 (−10.2 to 3.8)	−0.6 (−11.0 to 10.9)	−0.4 (−7.8 to 7.6)	2.2 (−10.0 to 16.0)	7.9 (−10.9 to 30.7)	−4.4 (−16.5 to 9.5)	−4.7 (−15.2 to 7.1)	1.6 (−14.9 to 21.4)	3.7 (−8.6 to 17.8)

Add age, sex, race	1.3 (−5.9 to 9.0)	2.1 (−7.7 to 12.9)	2.1 (−5.0 to 9.6)	5.0 (−8.2 to 20.0)	12.3 (−6.5 to 34.9)	−2.2 (−14.1 to 11.3)	−0.4 (−12.1 to 12.9)	0.4 (−15.4 to 19.1)	0.5 (−11.1 to 13.6)

All covariates^b^	1.4 (−5.9 to 9.2)	2.0 (−7.9 to 13.0)	1.6 (−5.4 to 9.2)	5.8 (−7.3 to 20.8)	13.0 (−5.8 to 35.5)	−4.2 (−15.7 to 9.0)	−0.4 (−11.9 to 12.6)	6.0 (−10.5 to 25.4)	−0.6 (−11.8 to 12.0)

Chicago (*n* = 753)									
PM (weather only)^a^	2.3 (−7.9 to 13.7)	6.1 (−3.8 to 17.1)	4.1 (−4.2 to 13.0)	4.3 (−4.5 to 13.9)	−6.5 (−13.9 to 1.6)	0.7 (−6.1 to 7.9)	−8.2 (−20.4 to 6.0)	−19.7 (−29.7 to −8.2)	−9.5 (−19.2 to 1.3)

Add age, sex, race	0.0 (−9.9 to 11.0)	6.3 (−4.1 to 17.8)	4.8 (−3.5 to 13.7)	1.8 (−6.6 to 10.9)	−0.7 (−8.8 to 8.1)	4.3 (−2.6 to 11.6)	−9.6 (−21.0 to 3.4)	−5.5 (−17.4 to 8.0)	−2.7 (−12.6 to 8.3)

All covariates^b^	−1.3 (−10.9 to 9.3)	4.9 (−5.2 to 16.1)	4.9 (−3.4 to 13.8)	2.0 (−6.4 to 11.2)	−0.8 (−8.9 to 8.1)	3.2 (−3.7 to 10.6)	−9.1 (−20.5 to 4.0)	−4.1 (−16.1 to 9.6)	−2.6 (−12.6 to 8.5)

Winston-Salem (*n* = 720)									
PM (weather/season only)^a^	−4.0 (−20.0 to 15.2)	−8.6 (−25.0 to 11.4)	−3.1 (−15.2 to 10.8)	11.5 (0.0 to 24.3)	5.3 (−6.5 to 18.6)	1.0 (−6.8 to 9.4)	−7.0 (−21.2 to 9.9)	−9.5 (−24.5 to 8.5)	−0.7 (−12.2 to 12.3)

Add age, sex, race	4.3 (−12.6 to 24.4)	−8.5 (−24.3 to 10.6)	−5.1 (−16.5 to 7.9)	5.6 (−4.3 to 16.6)	2.1 (−8.2 to 13.6)	0.6 (−6.4 to 8.1)	−15.3 (−27.6 to −1.0)	−11.9 (−25.6 to 4.3)	0.3 (−10.5 to 12.5)

All covariates^b^	1.9 (−14.2 to 21.2)	−8.7 (−24.1 to 9.8)	−5.0 (−16.2 to 7.8)	5.0 (−4.9 to 16.0)	2.0 (−8.3 to 13.5)	−0.5 (−7.5 to 7.0)	−13.9 (−26.3 to 0.7)	−11.1 (−24.8 to 5.0)	1.7 (−9.3 to 13.9)

St. Paul (*n* = 705)									
PM (weather/season only)^a^	0.0 (−9.6 to 10.7)	2.4 (−8.2 to 14.3)	−5.5 (−13.8 to 3.6)	4.8 (−2.9 to 13.1)	8.4 (−0.2 to 17.8)	−2.5 (−9.0 to 4.5)	−4.6 (−15.5 to 7.9)	3.4 (−9.4 to 18.0)	−4.5 (−14.5 to 6.7)

Add age, sex, race	−3.8 (−12.9 to 6.2)	−0.7 (−10.8 to 10.6)	−5.3 (−13.4 to 3.6)	4.9 (−1.9 to 12.2)	4.4 (−2.9 to 12.2)	−0.1 (−6.0 to 6.1)	−3.4 (−13.6 to 7.9)	−1.2 (−12.4 to 11.4)	−1.4 (−10.8 to 9.0)

All covariates^b^	−6.6 (−15.3 to 3.0)	−2.6 (−12.4 to 8.4)	−5.7 (−13.7 to 3.0)	5.1 (−1.8 to 12.5)	4.0 (−3.3 to 11.9)	−0.4 (−6.3 to 6.0)	−3.8 (−13.9 to 7.5)	−2.3 (−13.3 to 10.1)	−3.0 (−12.2 to 7.2)

Los Angeles (*n* = 683)									
PM (weather/season only)^a^	9.8 (−0.6 to 21.2)	6.2 (−4.4 to 18.0)	0.8 (−6.7 to 8.7)	7.6 (1.1 to 14.6)	6.9 (0.0 to 14.3)	0.5 (−4.2 to 5.5)	3.4 (−5.9 to 13.6)	2.3 (−7.5 to 13.1)	1.5 (−5.6 to 9.1)

Add age, sex, race	5.3 (−5.2 to 16.9)	1.2 (−9.0 to 12.5)	−1.6 (−8.5 to 5.9)	4.4 (−1.9 to 11.1)	5.2 (−1.2 to 12.1)	1.5 (−2.8 to 6.0)	−2.6 (−11.3 to 7.0)	−0.9 (−9.9 to 8.9)	2.7 (−3.8 to 9.6)

All covariates^b^	5.5 (−4.8 to 17.0)	1.1 (−8.9 to 12.2)	−2.2 (−8.9 to 5.1)	4.4 (−2.0 to 11.2)	5.2 (−1.3 to 12.1)	1.2 (−3.2 to 5.7)	−4.3 (−12.8 to 5.0)	−3.3 (−12.0 to 6.1)	1.3 (−5.0 to 8.0)

Baltimore (*n* = 560)									
PM (weather/season only)^a^	33.3 (−0.6 to 78.8)	22.5 (−6.7 to 60.8)	−3.9 (−17.4 to 11.8)	−21.2 (−34.5 to −5.4)	−15.6 (−28.8 to 0.1)	−3.8 (−12.5 to 5.8)	5.7 (−19.8 to 39.4)	16.0 (−10.2 to 49.9)	−8.9 (−21.0 to 5.1)

Add age, sex, race	30.5 (−2.8 to 75.4)	19.0 (−9.8 to 57.0)	−3.3 (−16.6 to 12.0)	−18.8 (−32.0 to −3.1)	−11.7 (−25.2 to 4.2)	−2.3 (−10.6 to 6.8)	24.6 (−4.5 to 62.5)	38.0 (7.8 to 76.6)	−5.9 (−17.6 to 7.4)

All covariates^b^	40.6 (5.2 to 87.9)	24.2 (−5.4 to 63.0)	−6.1 (−18.8 to 8.5)	−17.1 (−30.9 to −0.7)	−12.4 (−26.0 to 3.7)	−1.1 (−9.6 to 8.2)	28.1 (−2.0 to 67.4)	37.7 (7.4 to 76.5)	−3.9 (−15.9 to 9.9)

Regression results are reported as percent differences (95% CI) in outcome variables [100 × (exponentiated mean difference − 1)]. A positive point estimate for YM indicates greater arterial stiffness with higher exposure; a negative point estimate for C_1_ and C_2_ indicates reduced compliance with higher exposure. PM levels are from nearest monitor or space–time model (see Table 2).

a Weather and season terms included in all models: apparent temperate spline with 3 degrees of freedom; and indicator variables to represent months with average monthly temperature above 10°C (50°F) during baseline recruitment period (all year for Los Angeles, May–October for Chicago and St. Paul, April–October for remaining three sites).

b All covariates for YM are age, sex, race, fasting blood glucose, triglycerides, diabetes, seated MAP, smoking status, pack-years, cigarettes per day, ETS exposure, physical activities, waist:hip ratio, season, and weather. For C_1_ and C_2_, height, weight, and heart rate also were included.

**Table 6:** Percent difference (95% CI) in three arterial stiffness outcomes per 10-μg/m^3^ increment in 2001 PM exposure under various covariate adjustment schemes, stratified by study site: MESA, 2000–2002.

	YM		C_1_		C_2_	
Outcome/covariates	PM_10_	PM_2.5_	PM_10_	PM_2.5_	PM_10_	PM_2.5_
New York (*n* = 575)						
PM (weather/season only)^a^	−0.1 (−0.4 to 0.2)	−0.9 (−2.9 to 1.1)	−0.1 (−0.4 to 0.2)	−0.9 (−2.9 to 1.1)	−0.4 (−0.9 to 0.1)	0.6 (−2.8 to 4.2)
Add age, sex, race	0.0 (−0.3 to 0.2)	−0.4 (−2.3 to 1.5)	0.0 (−0.3 to 0.2)	−0.4 (−2.3 to 1.5)	−0.2 (−0.7 to 0.3)	0.9 (−2.5 to 4.5)
All covariates^b^	0.0 (−0.3 to 0.3)	−0.3 (−2.2 to 1.6)	0.0 (−0.3 to 0.3)	−0.3 (−2.2 to 1.6)	−0.2 (−0.7 to 0.3)	0.8 (−2.6 to 4.3)

Chicago (*n* = 753)						
PM (weather/season only)^a^	0.7 (−0.1 to 1.4)	0.9 (−1.9 to 3.7)	−0.7 (−1.4 to −0.1)	−2.6 (−4.8 to −0.3)	−0.2 (−1.2 to 0.9)	−0.4 (−4.0 to 3.4)
AAdd age, sex, race	0.9 (0.1 to 1.6)	1.7 (−1.0 to 4.5)	−0.5 (−1.1 to 0.1)	−1.5 (−3.7 to 0.8)	−0.3 (−1.3 to 0.7)	0.8 (−2.7 to 4.5)
All covariates^b^	0.7 (0.0 to 1.5)	1 (−1.7 to 3.8)	−0.6 (−1.2 to 0.1)	−1.8 (−4.0 to 0.5)	−0.2 (−1.2 to 0.8)	1 (−2.5 to 4.7)

Winston-Salem (*n* = 720)						
PM (weather/season only)^a^	−0.1 (−3.0 to 3.0)	−0.2 (−5.1 to 5.0)	1.1 (−0.7 to 2.9)	0.6 (−2.4 to 3.8)	1.3 (−1.5 to 4.1)	−0.8 (−5.4 to 3.9)
Add age, sex, race	0.7 (−2.3 to 3.7)	−2.0 (−6.7 to 3.0)	1.5 (−0.2 to 3.2)	0.6 (−2.2 to 3.4)	0.5 (−2.2 to 3.1)	0.5 (−3.8 to 5.1)
All covariates^b^	0.6 (−2.3 to 3.5)	−0.3 (−5.0 to 4.6)	1.6 (0.0 to 3.3)	0.6 (−2.1 to 3.5)	0.1 (−2.4 to 2.8)	0.7 (−3.6 to 5.2)

St. Paul (*n* = 705)						
PM (weather/season only)^a^	−0.8 (−1.8 to 0.2)	0.6 (−2.8 to 4.2)	−0.2 (−0.9 to 0.6)	0.2 (−2.4 to 2.9)	−0.5 (−1.7 to 0.7)	1.2 (−2.9 to 5.5)
Add age, sex, race	−0.7 (−1.7 to 0.3)	1.5 (−1.9 to 5.0)	0.2 (−0.4 to 0.9)	−1.5 (−3.7 to 0.8)	−0.1 (−1.2 to 1.0)	−1.5 (−5.2 to 2.3)
All covariates^b^	−0.7 (−1.7 to 0.3)	1.7 (−1.6 to 5.2)	0.2 (−0.5 to 0.9)	−1.6 (−3.8 to 0.7)	−0.2 (−1.3 to 0.9)	−1.6 (−5.3 to 2.2)

Los Angeles (*n* = 683)						
PM (weather/season only)^a^	1.2 (−0.3 to 2.7)	0.4 (−0.7 to 1.5)	0.9 (−0.1 to 1.8)	−0.7 (−1.4 to −0.1)	1.2 (−0.2 to 2.7)	−0.7 (−1.8 to 0.3)
Add age, sex, race	−0.1 (−1.9 to 1.8)	0.2 (−0.9 to 1.4)	0.2 (−0.9 to 1.3)	−0.4 (−1.1 to 0.3)	0.2 (−1.4 to 1.9)	−0.1 (−1.1 to 0.9)
All covariates^b^	0.0 (−1.7 to 1.8)	0.0 (−1.1 to 1.1)	0.1 (−1.0 to 1.2)	−0.4 (−1.1 to 0.3)	0.2 (−1.4 to 1.8)	−0.3 (−1.3 to 0.7)

Baltimore (*n* = 560)						
PM (weather/season only)^a^	1.1 (−0.4 to 2.7)	1.8 (−2.2 to 6.0)	−1.3 (−2.3 to −0.3)	−0.5 (−3.0 to 2.0)	−1.2 (−2.7 to 0.3)	0.6 (−3.1 to 4.5)
Add age, sex, race	0.7 (−1.0 to 2.4)	1 (−2.9 to 5.0)	−0.8 (−1.8 to 0.2)	0.2 (−2.1 to 2.7)	0.2 (−1.3 to 1.7)	1.5 (−2.0 to 5.2)
All covariates^b^	0.7 (−0.9 to 2.4)	2.3 (−1.6 to 6.4)	−0.6 (−1.6 to 0.4)	0.6 (−1.9 to 3.0)	0.5 (−1.0 to 2.0)	2.3 (−1.3 to 6.1)

Regression results reported as percent differences (95% CI) in outcome variables [100 × (exponentiated mean difference − 1)]. A positive point estimate for YM indicates greater arterial stiffness with higher exposure; a negative point estimate for C_1_ and C_2_ indicates reduced compliance with higher exposure. Yearly average of PM concentrations taken from monitor nearest the MESA participant’s residence in 2001.

a Weather and season terms included in all models: apparent temperate spline with 3 degrees of freedom, interacted with site; and indicator variables to represent months with average monthly temperature above 10°C (50°F) during baseline recruitment period (all year for Los Angeles; May–October for Chicago and St. Paul; April–October for remaining three sites).

b All covariates for YM are age, sex, race, fasting blood glucose, triglycerides, diabetes, seated MAP, smoking status, pack-years, cigarettes per day, ETS exposure, physical activities, waist:hip ratio, season, and weather. For C_1_ and C_2_, in addition, height, weight, and heart rate were included.
